# Pharmazie Reloaded – Umfassende Kompetenz rund um das Arzneimittel

**DOI:** 10.1007/s00103-025-04047-z

**Published:** 2025-05-13

**Authors:** Ulrich Jaehde, Peter Heisig

**Affiliations:** 1 Abteilung Klinische Pharmazie, Pharmazeutisches Institut, Rheinische Friedrich-Wilhelms-Universität Bonn, An der Immenburg 4, 53121 Bonn, Deutschland; 2 Pharmazeutische Biologie und Mikrobiologie, Institut für Biochemie und Molekularbiologie, Universität Hamburg, Bundesstraße 45, 20146 Hamburg, Deutschland

Gemäß § 1 Bundes-Apothekerordnung sind Apothekerinnen und Apotheker „berufen, die Bevölkerung ordnungsgemäß mit Arzneimitteln … zu versorgen“ und „damit der Gesundheit des einzelnen Menschen und des gesamten Volkes zu dienen“. Sie tragen damit Verantwortung für die Entwicklung, Herstellung, Prüfung und Qualität von Arzneimitteln, aber auch zunehmend für eine wirksame und sichere Arzneimitteltherapie zusammen mit anderen Heilberufen. Apothekerinnen und Apotheker üben ihren Beruf nicht nur in Apotheken, sondern überall dort aus, wo Arzneimittel eine Rolle spielen, wie z. B. in der pharmazeutischen Industrie und der öffentlichen Gesundheitsverwaltung. Damit sind sie *die* Expertinnen und Experten rund um das Arzneimittel, deren Kompetenz auf einer universitären Ausbildung und einer qualitätsgesicherten Fort- und Weiterbildung beruht.

Neue Arzneimittel und innovative Arzneimitteltherapien, aber auch sich stetig ändernde Bedürfnisse der Patientinnen und Patienten führen zu wachsenden Anforderungen an den Apothekerberuf. Aus‑, Fort- und Weiterbildung müssen mit diesen Entwicklungen Schritt halten. Dieses Themenheft möchte einen Überblick über die Historie, Gegenwart und Zukunftsperspektiven der Qualifizierung von Apothekerinnen und Apothekern geben.

Im ersten Beitrag beschreibt *Axel Helmstädter* die Entwicklung der Apothekerausbildung von einer handwerklich geprägten Lehre zu einem naturwissenschaftlich geprägten Studium, das zunehmend auch therapeutische Aspekte beinhaltet. In anderen Ländern sind patientenorientierte Inhalte schon länger ins Curriculum integriert als in Deutschland. Wie *Anita Weidmann* und *Freyja Jónsdóttir* in ihrem Beitrag zur Apothekerausbildung im internationalen Vergleich ausführen, ist dies vor allem auf den engen, durch die Approbationsordnung für Apotheker (AAppO) vorgegebenen Rechtsrahmen in Deutschland zurückzuführen, der sich hemmend auf zeitnahe Anpassungen des Curriculums an neue Entwicklungen in den pharmazeutischen Wissenschaften auswirkt.

Aber wie sollte ein modernes und zukunftsfähiges Pharmaziestudium aussehen? *Bernd Clement* stellt in seinem Beitrag dar, dass die Naturwissenschaften auch in Zukunft die Basis bilden, um ein tieferes Verständnis für Arzneistoffe und deren Wirkung im Organismus entwickeln zu können. Gleichzeitig muss das Pharmaziestudium jedoch zu patientenorientiertem Arbeiten befähigen, damit die wissenschaftlichen Kenntnisse bei den Patientinnen und Patienten auch ankommen. *Frank Dörje et al.* stellen in ihrem Beitrag verschiedene patientenorientierte Lehrformate vor, die bereits in Deutschland eingesetzt werden. Da diese Lehrformate von der aktuell gültigen AAppO nicht gefordert werden, ist die Umsetzung an den deutschen Universitäten jedoch äußerst heterogen. Dies gilt auch für die Aufnahme interprofessioneller Lehrveranstaltungen in das Curriculum. Diese sind wichtig, damit die verschiedenen Berufsgruppen, vor allem Pharmazie- und Medizinstudierende, interprofessionelle Zusammenarbeit bereits im Studium erlernen, wie *Jennifer Weber et al.* in ihrem Beitrag herausarbeiten. Die fortschreitende Digitalisierung bietet weitere Möglichkeiten, das Pharmaziestudium weiterzuentwickeln. *Christoph Ritter* stellt in seinem Artikel eine Reihe von digitalen Lernmethoden vor und welche Erfahrungen damit bereits im Pharmaziestudium gemacht wurden.

Aus den oben genannten Gründen ist es nicht überraschend, dass bereits seit vielen Jahren intensiv über eine Novellierung der AAppO diskutiert wird. Die Studierenden haben hierzu eine klare Haltung, wie *Elisabeth Jones* und *Nikita Vassiljev* in ihrem Beitrag für den Bundesverband der Pharmaziestudierenden in Deutschland e. V. (BPhD) ausführen. Neben einer umfassenden Novellierung der AAppO schlagen sie einen Nationalen Kompetenzorientierten Lernzielkatalog Pharmazie (NKLP) vor, der einfacher an die sich wandelnden Anforderungen angepasst werden kann als die AAppO. Wie *Berit Winter* in ihrem Beitrag vorstellt, wurde im Jahr 2017 unter Federführung der Bundesapothekerkammer ein „Kompetenzorientierter Lernzielkatalog Pharmazie – Perspektivpapier Apotheke 2030“ (KLP-P) verabschiedet, der aufzeigt, wie die Gestaltungsspielräume in der aktuell gültigen AAppO genutzt werden können, um notwendige Ausbildungsinhalte und Kompetenzen einzuführen bzw. zu intensivieren. In den letzten zwei Jahrzehnten haben sich die apothekerlichen Aufgaben und Tätigkeitsschwerpunkte jedoch derart gewandelt, dass eine Novellierung der AAppO unausweichlich geworden ist. Dies betrifft auch die Notwendigkeit, neue Lehrinhalte, einschließlich der dafür benötigten wissenschaftlichen Grundlagen, zeitnah in die akademische Ausbildung zu integrieren. Nur aufbauend auf einem solchen Fundament lassen sich die geforderten, dem aktuellen Stand der Wissenschaft angepassten Kompetenzen zur Entwicklung, Qualitätssicherung und patientenorientierten Anwendung von Arzneimitteln vermitteln. Besonders deutlich wird dieser Aspekt im Bereich der Lebenswissenschaften, deren enormer Zuwachs an molekularen Grundlagenkenntnissen sich zunehmend auf die Inhalte aller pharmazeutischen Fachdisziplinen auswirkt. An einem runden Tisch erarbeiteten deshalb bereits 2022 die Bundesapothekerkammer, die pharmazeutische Hochschullehrerschaft, apothekerliche Fachorganisationen und die Studierendenvertretung gemeinsam ein Positionspapier mit einem konkreten Vorschlag zur Novellierung der Approbationsordnung, das in einen Antrag an das Bundesministerium für Gesundheit (BMG) mündete.

Vor dem Hintergrund des ständig wachsenden pharmazeutischen Wissens kommt der Fort- und Weiterbildung von Apothekerinnen und Apothekern eine besondere Bedeutung zu. *Constanze Schäfer* beschreibt in ihrem Beitrag nicht nur die Entwicklung der Fort- und Weiterbildung in Deutschland, sondern zeigt auch zukünftige Perspektiven wie die kontinuierliche professionelle Kompetenzentwicklung (CPD) auf, die beispielsweise bereits im Vereinigten Königreich praktiziert wird. Konsequenterweise sollte dieses Konzept auch die in Aus‑, Fort- und Weiterbildung engagierten Lehrkräfte einschließen. Das interdisziplinäre Grundkonzept des Studiengangs Pharmazie mit Fokus auf das Arzneimittel bietet eine hervorragende Basis, die Lehre zur Entwicklung und Anwendung innovativer Arzneistoffe und Arzneiformen sowie neuer Therapieformen an konkreten Fragestellungen auszurichten. Die entsprechenden Inhalte können auf diese Weise aus unterschiedlichen fachlichen Perspektiven gelehrt werden und somit kann – entsprechend dem Titel des vorliegenden Heftes – eine „umfassende Kompetenz rund um das Arzneimittel“ vermittelt werden.

Abschließend bedanken wir uns herzlich bei allen Autorinnen und Autoren für ihre engagierten Beiträge zu diesem Heft. Es wird deutlich, wie fundiert und umfassend Apothekerinnen und Apotheker in allen Disziplinen rund um das Arzneimittel ausgebildet sind und wie wichtig eine kontinuierliche Aktualisierung der Aus‑, Fort- und Weiterbildungsinhalte für die Qualität der Arzneimittelversorgung ist. Wir hoffen, dass das Heft neue Impulse für die überfällige Anpassung der Apothekerausbildung in Deutschland gibt. Allen Leserinnen und Lesern wünschen wir viel Freude und Inspiration bei der Lektüre dieses Themenhefts.

